# Chemical and Biological Evaluation of Essential Oils from Two Species of Myrtaceae — *Eugenia uniflora* L. and *Plinia trunciflora* (O. Berg) Kausel

**DOI:** 10.3390/molecules16129827

**Published:** 2011-11-25

**Authors:** João Henrique G. Lago, Elisângela Dutra Souza, Bruna Mariane, Renata Pascon, Marcelo A. Vallim, Roberto Carlos C. Martins, Adriana A. Baroli, Bianca A. Carvalho, Marisi G. Soares, Roberta T. dos Santos, Patricia Sartorelli

**Affiliations:** 1 Instituto de Ciências Ambientais, Químicas e Farmacêuticas, Universidade Federal de São Paulo, Campus Diadema 09972-270, SP, Brasil; 2 Centro de Estudos Químicos, UNIFIEO, Centro Universitário FIEO, Osasco 06020-190, SP, Brasil; 3 Instituto de Química, Universidade Federal de Alfenas, Alfenas 37130-000, MG, Brasil

**Keywords:** *Eugenia uniflora* L., *Plinia trunciflora* (O. Berg) Kausel, essential oils, antimicrobial activity

## Abstract

The chemical composition and antimicrobial activity of essential oils obtained from leaves of two Myrtaceae species–*Eugenia uniflora* L. and *Plinia trunciflora* (O. Berg) Kausel–were determined. Analysis by GC/MS as well as determination of Kovatz indexes indicated atractylone (26.78%) and curzerene (17.96%) as major constituents of *E. uniflora* oil and α-cadinol (19.15%), apiole (11.15%) and cubenol (5.43%) as main components in *P. trunciflora* oil. Both essential oils were tested for antimicrobial activity against yeasts and bacteria. *E. uniflora* and *P. trunciflora* essential oils were active towards two Gram-positive bacteria, *Streptococcus equi* and *Staphylococcus* *epidermis*. In addition, biological activity of both essential oils was detected for pathogenic yeasts of the genus *Candida* and *Cryptococcu*s. *E. uniflora* was active towards all yeast tested and exhibited interesting minimal inhibitory concentrations (0.11 to 3.75 mg/mL) across a broad spectrum of activity.

## 1. Introduction

The Myrtaceae comprise 4,620 species distributed in 140 genera whose occurrence has been described in tropical and subtropical regions of the World, mainly Australia and Central and South America [1]. The main genera are *Myrtus*, *Psidium*, *Pimenta*, *Eugenia*, *Pseuocaryophyllus*, *Syzygium*, *Eucalyptus*, *Leptospermum*, *Plinia*, and *Malaleuca*. Several members of this family are used in folk medicine as antidiarrheal, antimicrobial, antioxidant, antirheumatic, anti-inflammatory, and cleansing agents, and they are also used to decrease blood cholesterol [2]. Chemically, several members mainly accumulate flavonoids, tannins and other phenolic derivatives [3,4,5]. In addition, this family represents an important source of essential oils with biological activities ranging from bacteriostatic and fungistatic to anti-inflammatory [6], which have been used as antimicrobial and antifungal agents in creams, soaps, and toothpastes [7].

The chemical composition of essential oils from several species of *Eugenia* was previously described in the literature, especially those from *E. uruguayensis* Camb. [8], *E. banderensis* Urb. [9], *E. nitida* Camb. [10], *E. brasiliensis* Lam. [11], and *E. uniflora* L. [12,13,14]. Sesquiterpenes (hydrocarbon and oxygenated derivatives) were found as the main class of volatile constituents possessing antibacterial, antifungal, anti-inflammatory, and cytotoxic activities [6]. In addition to these derivatives, monoterpenes and phenylpropanoids were also described in these oils.

*Eugenia uniflora* L., popularly known as “pitangueira”, is a highly appreciated species in Brazil due to its reddish fruits with a sweet taste, and tea obtained from its leaves has been used in folk medicine against fever, stomach diseases, hypotension, and gout, and as a hypoglycemiant [15,16]. *Plinia* is another genus of Myrtaceae that shows several traditional uses and is also a source of essential oils composed mainly of sesquiterpenes [17,18]. *Plinia trunciflora* (O. Berg) Kausel, a synonym of *MyrciariatruncifloraI* Mart. (O. Berg), is popularly named “jabuticabeira” in Brazil and its edible fruits are also appreciated. In folk medicine, species of *Plinia* have been used in the treatment of stomach disorders, throat afflictions, and diabetes [19]. Biological properties of essential oil from *P. trunciflora* have not been reported, but leaf extract showed antioxidant and antimicrobial activities [20]. In a continuation of our systematic studies on pharmacologically active volatiles from Brazilian plants [21,22] we describe in this paper an evaluation of the chemical composition and antimicrobial activity of essential oils from leaves of *E. uniflora* and, for the first time, antimicrobial activity of essential oil from the leaves of *P. trunciflora*.

## 2. Results and Discussion

Hydrodistillation of the fresh leaves from *E. uniflora* afforded a yellow viscous oil with a pungent odor (yield 0.1% w/w), while *P. trunciflora* provided an odorless and colorless oil (yield 0.02% w/w). The constituents of essential oil of *E. uniflora* and *P*. *trunciflora* were analyzed by GC-FID-MS followed by calculation of Kovatz indexes. In total, 56 compounds were identified (Table 1) accounting for 77.16 and 72.92% of the total oil composition of *E. uniflora* and *P.* *trunciflora*, respectively. In both oils, oxygenated sesquiterpenes were the major constituents (*E. uniflora*: 49.89%; *P. trunciflora*: 48.09%), but hydrocarbon sesquiterpenes (24.79%) were also identified in *E. uniflora*. Monoterpenes were present in smaller concentrations in both oils, but hydrocarbon monoterpene occurrence was restricted to *E. uniflora* (2.32%), while oxygenated derivatives were dominant in *P*. *trunciflora* (13.34%). In addition, phenylpropanoids were present in lower concentrations in *E. uniflora* (0.09%) and in higher amounts in *P. trunciflora*, mainly due to the presence of apiole (11.15%).

**Table 1:** Chemical composition of essential oil from leaves of *E. uniflora* and *P. trunciflora*.

Compound	KI	*E. uniflora*	*P. trunciflora*
β-pinene	980	0.22	
α-phellandrene	1005	0.05	-
*ortho*-cymene	1022	0.09	-
sylvestrene	1027	0.08	-
( *Z*)-β-ocimene	1040	0.51	-
( *E*)-β-ocimene	1050	1.24	-
γ-terpinene	1062	0.05	-
terpinolene	1088	0.08	-
linalool	1098	0.04	-
*neo*-menthol	1165	-	1.86
hexenyl butyrate	1186	0.03	-
α-terpineol	1189	-	7.48
n-dodecane	1199	-	0.36
*trans*-pulegol	1213	-	0.48
anethole	1251	0.09	-
isobornyl acetate	1285	-	0.41
δ-elemene	1339	0.16	-
α-copaene	1376	0.08	
γ-elemene	1433	0.22	-
α-humulene	1454	0.25	-
γ-muurolene	1477	3.59	-
β-selinene	1485	0.21	-
viridiflorene	1493	0.08	-
curzerene	1496	17.96	-
α-muurolene	1499	0.12	-
α-bisabolene	1504	1.35	-
δ-cadinene	1524	0.50	-
α-cadinene	1538	0.09	-
selina-3,7(11)-diene	1542	0.18	-
elemol	1549	0.02	-
germacrene B	1556	9.31	-
ledol	1565	-	1.80
caryophyllene alcohol	1568	0.72	-
spathulenol	1576	1.08	-
viridiflorol	1590	3.08	2.74
longiborneol	1592	2.09	-
10- *epi*-γ-eudesmol	1619	-	0.48
3- *iso*-thujopsanone	1637	0.27	-
*epi*-α-cadinol	1640	0.41	3.29
*epi*-α-muurolol	1641	1.37	-
cubenol	1641	0.44	5.43
α-muurolol	1645	-	2.82
vulgarone B	1647	-	2.65
atractylone	1653	26.78	-
α-cadinol	1653	-	19.15
valerianol	1655	-	0.85
5-hydroxyisobornyl isobutyrate	1655	-	2.75
valeranone	1672	-	2.78
apiole	1680	-	11.15
*iso*-longifolol	1726	-	1.28
coniferyl alcohol ( *E*)	1729	-	1.84
zerumbone	1731	4.18	-
6R,7R-bisabolone	1737	0.11	-
α-sinensal	1752	-	0.88
8-cedren-13-ol acetate	1795	0.03	-
nootkatone	1800	-	2.14
*Hydrocarbon monoterpenes*		*2.32*	*-*
*Oxygenated monoterpenes*		*0.04*	*13.34*
*Hydrocarbon sesquiterpenes*		*24.79*	*-*
*Oxygenated sesquiterpenes*		*48.89*	*48.03*
*Phenylpropanoids*		*0.09*	*11.15*
*Other compounds*		*0.03*	*0.36*
**TOTAL**		**77.16**	**72.92**

Although the analyzed oils were composed mainly of sesquiterpenes, only three components were detected in both *E. uniflora* and *P. trunciflora* oils: Viridiflorol (3.08 and 2.74%), *epi*-α-cadinol (0.41 and 3.29%), and cubenol (0.44 and 5.43%). Atractylone (26.78%) was the main component of essential oil from *E. uniflora*, followed by curzerene (17.96%), which was responsible for the pungent odor detected in the analyzed oil [6]. Essential oils from *E. uniflora* leaves collected in several localities were previously studied and, despite variation in their chemical compositions, the predominance of sesquiterpenes was confirmed, except in an Argentinean specimen rich in monoterpenes [23]. Moreover, atractylone and curzerene (a Cope rearrangement product of the former), were detected as the main components from other studied specimens of *E. uniflora* [24,25,26]. Surprisingly, selina-1,3,7(11)-trien-8-one was previously found to be a main constituent of essential oils from leaves of four species of *E. uniflora* [25,27,28,29], but was not detected in the present work.

Analysis of the essential oil of *P. truncifolia* showed that 19.15% of the total constitution was due to the oxygenated sesquiterpene α-cadinol. Other major identified components (>5%) were apiole (11.15%), α-terpineol (7.48%), and cubenol (5.43%). Despite the chemical composition of essential oil from leaves of *P. trunciflora* already being described from a specimen collected in Southern Brazil, the obtained results showed some important differences, since in the previous study spathulenol and caryophyllene oxide were identified as the main components [17].

*Antimicrobial Activity*. To confirm the results found by this semi-quantitative method, a minimum inhibitory concentration (MIC) assay was applied to all positive strains. Table 2 presents the relevant data for the disk diffusion assay expressed as inhibition zone (IZ) and MICs for those strains sensitive to the presence of the two separate essential oils, where numbers in parenthesis represent the average percentage inhibition and standard deviation of three repetitions. For comparison, chloramphenicol (bacteria) and fluconazole (yeast) were used as standards in the disk diffusion assay (MICs ranged from 0.006–0.400 mg/mL for all tested microorganisms, as showed in Table 2). According to the literature [30], the minimum inhibitory concentration of fluconazole for *Candida spp*., *Cryptoccocus spp*. and *S. cerevisiae* ranges from 0.00012 to 0.064, 0.00025 to 0.032 and 0.00012 to 0.016 mg/mL, respectively. Variations in MICs may depend upon genetic variation among strains. As shown in Table 2, our results were consistent with the literature, which guaranteed that our MICs were a reliable tool to demonstrate the biological activity of both essential oils.

**Table 2:** Antimicrobial activity of two essential oils (from *E. uniflora* and *P. trunciflora*) evaluated by the disk diffusion method (IZ) and minimal inhibitory concentration (MIC).

Strains	*Eugenia uniflora*		*Plinia trunciflora*		Positive control
	IZ	MIC	IZ	MIC	MIC
	(mm)	(mg/mL)	(mm)	(mg/mL)	(mg/mL)
*S. equi*	1.4	7.50 (55 ± 6%)	1.4	0.12 (94 ± 3%)	0.025 **
*S. epidermidis*	1.6	7.50 (87 ± 2%)	1.6	0.12 (95 ± 4%)	0.400 **
*C. dubliniensis*	1.4	0.23 (93 ± 22%)	1.2	0.06 (99 ± 5%)	0.006 *
*C. tropicalis*	1.4	0.90 (85 ± 1%)	-	-	0.050 *
*C. albicans*	1.4	1.80 (93 ± 1%)	1.4	0.06 (82 ± 6%)	0.025 *
*C. glabrata*	1.6	0.93 (85 ± 8%)	1.4	0.12 (100 ± 0%)	0.050 *
*C. parapsilosis*	1.4	3.75 (85 ± 1%)	1.4	0.12 (98 ± 5%)	0.006 *
*C. grubii* (serotype A)	1.6	0.45 (89 ± 2%)	1.6	0.12 (97 ± 7%)	0.013 *
*C. gattii* (serotype C)	1.6	1.80 (75 ± 21%)	1.2	0.12 (98 ± 4%)	0.025 *
*C. gattii* (serotype B)	1.6	0.22 (99 ± 3%)	-	-	0.006 *
*C. neoformans* (serotype D)	1.6	0.11 (87 ± 13%)	-	-	0.006 *
*S. cerevisiae*	1.6	0.22 (91 ± 12%)	1.6	0.12 (100 ± 0%)	0.013 *
Negative control	1.0	NA	1.0	NA	NA

Numbers in parenthesis represent the average percentage inhibition (three repetitions) and standard deviation at each MIC; Legend: * fluconazole; ** chloramfenicol; - no biological activity detected; NA, does not apply.

None of the Gram-negative strains tested were inhibited, but two Gram-positive strains (*S. equi* and *S. epidermidis*) showed growth reduction in the presence of the essential oils. This was in accordance with several other reports where essential oils were shown to be more active towards Gram-positive than Gram-negative bacteria [31,32,33,34]. On the other hand, several species of opportunistic pathogens belonging to *Candida* and *Cryptoccocus* genus and the model organism *S. cerevisiae* were inhibited by the agents under investigation. Regarding *E. uniflora* oil, all yeasts except *C. krusei* were inhibited by smaller amounts of crude essential oil (0.11 to 1.80 mg/mL) than *C. parapsilosis*, which required higher concentrations of oil for growth inhibition (3.75 mg/mL). Furthermore, *P. trunciflora* displayed a remarkable effect, except for *C. tropicalis*, *C. krusei* and two *Cryptoccocus* serotypes (C and D), which did not show any growth inhibition.

Regarding the genus *Candida*, Sokmem *et al*. [35] reported no antimicrobial activity against *C. krusei* using methanol extracts of *Achillea sintenisii*, while the essential oil of this plant showed mild biological activity against *C. albicans* and *C. krusei*.

Comparison of the two essential oils revealed that the oil from *P. trunciflora* was more efficient against *C. albicans* than oil obtained from *E. uniflora* leaves (0.06 and 1.8 mg/mL). This might be of great pharmaceutical interest because this continues to be the leading cause of disease in immunodeficient patients [36]. A similar profile was detected for *C. grubbii* (MICs of 0.12 and 0.45 mg/mL for *P. trunciflora* and *E. uniflora*, respectively), which is the major pathogen in fungal meningitis [37].

*E. uniflora* and *P. trunciflora* oils also demonstrated remarkable biological activity against *S. cerevisiae* (0.22 and 0.12 mg/mL, respectively) and *C. neoformans* (0.45 and 0.12 mg/mL, respectively). From a clinical point of view, these results could be important as these essential oils may represent an alternative to antimicrobial treatment. Furthermore, all three yeasts tested are great model pathogens, indicating that they can be employed in genetic studies that will shed some light on the mechanism of action of essential oils leading to growth inhibition. For example, Tamae *et al*. [38] used a collection of 4,000 *E. coli* knockout mutants to uncover the genetic basis of different antibiotic actions. These authors reported that the absence of 140 genes caused sensitivity to 7 different antimicrobial substances. The same kind of study could be done with *S. cerevisiae*, *C. neoformans* and *C. albicans* because they all have full genome sequences and knockout collections available.

## 3. Experimental

### 3.1. Plant Material

Leaves of *Eugenia uniflora* and *Plinia trunciflora* were collected in Osasco City, São Paulo State, Brazil (349966-W/7536935-N) in May 2010. Voucher specimens were compared with those under number SPF 195596/HRCB 51587 deposited in the Herbarium of Parque Ecológico da Pavuna, Botucatu SP, Brazil.

### 3.2. Essential Oil Extraction and Analysis

Fresh leaves (200 g) of *E. uniflora* and *P. trunciflora* were individually extracted over five hours by steam distillation in a Clevenger type apparatus to afford the crude essential oils (*E*. *uniflora:* 400 mg and *P. trunciflora*: 20 mg). The oils were then analyzed by GC and GC-MS and the identification of the individual compounds was achieved by comparison of retention indexes (determined relative to the retention times of a series of *n*-alkanes) on a non-polar column and recorded mass spectra with those available in the system [39]. GC chromatograms were obtained on a Shimadzu GC-2010 gas chromatograph equipped with an FID-detector and an automatic injector (Shimadzu AOC-20i) using a RtX-5 capillary column (5% phenyl, 95% polydimethylsiloxane, 30 m × 0.32 mm × 0.25 μm film thickness, Restek, USA). These analyses were performed by injecting 1.0 μL of a 1.0 mg/mL solution of volatile oil in CH_2_Cl_2_ in a split mode (1:30) employing helium as the carrier gas (1 mL/min) under the following conditions: injector and detector temperatures of 220 °C and 250 °C, respectively; oven programmed temperature from 40–240 °C at 3 °C/min, holding 5 min at 240 °C. The percentage compositions of the oil samples were computed by internal normalization from the GC peak areas without using correction for response factors. GC/MS analysis was conducted in a Shimadzu GC-17A chromatograph interfaced with a MS-QP-5050A mass spectrometer. Helium was used as the carrier gas. The MS operating conditions were an ionization voltage of 70 eV and an ion source temperature of 230 °C with the same conditions described above. 

### 3.3. Microbial Strains

To test the antimicrobial activity of essential oil from leaves of *E. uniflora* and *P. trunciflora*, Gram-positive, Gram-negative and yeast strains were submitted to a disk diffusion assay. Thus three different dilutions (1:10, 1:20 and 1:50) of the essential oils from *E.uniflora* and *P*. *trunciflora* were tested against seventeen microbial strains, which sources were reported in Table 3.

**Table 3:** Target strains used for antimicrobial activity assays.

Species	Designation
Bacteria	
*Escherichia coli*	-
*Serratia marcescens*	CBMAI 469
*Pseudomonas aeruginosa*	CBMAI 602
*Streptococcus equi*	CBMAI 264
*Staphylococcus epidermidis*	CBMAI 604
Yeast	
*Candida dubliniensis*	ATCC 7978
*Candida tropicalis*	ATCC 13803
*Candida albicans*	ATCC 18804
*Candida glabrata*	ATCC 90030
*Candida parapsilosis*	Clinical isolate 68
*Candida krusei*	Clinical isolate 9602
*Candida albicans*	CBMAI 560
*Cryptococcus grubii*	KN99 (serotype A)
*Cryptococcus gattii*	NIH312 (serotype C)
*Cryptococcus gattii*	R265 (serotype B)
*Cryptococcus neoformans*	JEC21 (serotype D)
*Saccharomyces cerevisiae*	BY4742

### 3.4. Media, Antibiotics and Growth Conditions

Yeast were cultivated on agar plates containing YEPD (1% yeast extract, 2% peptone, 2% dextrose and 2% agar) or RPMI1640 (Sigma). Gram-negative bacteria were grown in LB (0.5% yeast extract, 1% tryptone, 1% NaCl and 2% agar) and Gram-positive bacteria were tested in BHI (Himedia). Fluconazole (Sigma) and hygromycin B (Invitrogen) were used as positive controls for yeast and chloramphenicol (Sigma) was the positive control for bacteria. Essential oils were diluted in DMSO or saline (0.9%) plus Tween 80 (0.5%).

### 3.5. Disk Diffusion Assay

Antimicrobial activity was initially evaluated by the disk diffusion method according to the National Committee for Clinical Laboratory Standards (NCCLS, M26-T). Thin agar plates were prepared with 10 mL of YEPD (yeast), LB (Gram negative) and BHI (Gram positive) media. Three milliliters of liquid cultures were grown at 30 °C with aeration (150 rpm) overnight on YEPD (yeast), LB (Gram-negative) or BHI (Gram positive). A top agar was prepared by mixing each culture (100 µL) with soft agar medium for confluent plates (YEPD, LB or BHI plus 1% agar, 10 mL) and poured on top of the thin agar (2% agar). Sterilized 5 mm filter paper disks were then impregnated with essential oils diluted in DMSO (20 µL). The disks were placed on top of agar plates and incubated at 30 °C for 24 or 48 hours depending on the microorganism. Hygromycin (1 mg) and chloramphenicol (200 µg) were used as positive controls for yeast and bacteria, respectively. Negative control was prepared by impregnating the paper disks with the same amount of DMSO used to dilute the essential oils. All tests were performed in triplicate. The inhibition zone (IZ) was determined by measuring the whole halo diameter divided by the disk size (5 mm).

*Minimum inhibitory concentration*. Microdilution tests were conducted in sterile 96 well micro titer plates in a total volume of 100 µL according to the National Committee for Clinical Laboratory Standards (NCCLS, M100-S9). Microorganisms were cultured in test tubes overnight at 30 °C in 3 mL medium (RPMI 1640 for yeast and BHI for bacteria) in a rotary shaker (150 rpm). The cultures were diluted and adjusted to 1–2 × 10^2^ CFU/mL, which was confirmed by viability counts on YEPD and BHI plates (100 µL of diluted cells). Essential oils and reference standards were then serial diluted two-fold and tested. A sterilization control containing medium only (negative control) and growth control containing cell and DMSO (10 µL) or saline (10 µL) and Tween 80 were included as controls. Micro titer plates were then incubated at 30 °C for 24 or 48 hours depending on the microorganism. Finally, the absorbance at 530 nm was measured in a plate reader (Logen, MT-960) and the minimum inhibitory concentration was considered the lowest concentration at which at least 80% of growth was inhibited. All tests were performed in triplicate. The concentration range for each agent was as follows: *E. uniflora* 7.5–0.11 mg/mL; *M. trunciflora* 0.12–0.06 mg/mL; fluconazole 0.05–0.0007 µg/mL and chloramphenicol 0.400–0.00312 mg/mL.

## 4. Conclusions

Chemically, the essential oils from leaves of *E. uniflora* and *P. trunciflora* showed qualitative differences in respect to identified monoterpenes, sesquiterpenes, and phenylpropanoids. Both analyzed oils displayed interesting antimicrobial activities against several Gram-positive bacteria, mainly *S. equi* and *S. epidermidis*. Additionally, *E. uniflora* essential oil showed activity against all yeast strains tested, but primarily for *C. gattii* and *C. neoformans*, while *P. trunciflora* oil was active against *C. dubliniensi* and *C. albicans*. These results suggested that the observed activity might be related to the specific composition of sesquiterpenes in the oils. The obtained data clearly indicated that the essential oils of these two species of Myrtaceae could be exploited as antibacterial and fungicide agents.

## References

[1] Mabberly D.J. (1997). The Plant Book. A Portable Dictionary of the Vascular Plants.

[2] di Stasi L.C., Hiruma-Lima C.A. (2002). Plantas Medicinais na Amazônia e na Mata Atlântica.

[3] Kuskoski E.M., Vega J.M., Rios J.J., Fett R., Troncoso A.M., Asuero A.G. (2003). Characterization of anthocyanins from the fruits of baguaçu (*Eugenia umbelliflora* Berg). J. Agric. Food Chem..

[4] Fischer L.G., Santos D., Serafin C., Malheiros A., Delle Monache F., Delle Monache G., Cechinel Filho V., de Souza M.M. (2008). Further antinociceptive properties of extracts and phenolic compounds from *Plinia glomerata* (Myrtaceae) leaves. Biol. Pharm. Bull..

[5] Nuengchamnong N., Ingkaninan K. (2009). On-line characterization of phenolic antioxidants in fruit wines from family Myrtaceae by liquid chromatography combined with electrospray ionization tandem mass spectrometry and radical scavenging detection. LTW-Food Sci. Technol..

[6] Stefanello M.E.A., Pascoal A.C.R.F., Salvador M.J. (2011). Essential oils from neotropical myrtaceae: Chemical diversity and biological properties. Chem. Biodivers..

[7] Lis-Balchin M., Hart S.L., Deans S.G. (2000). Pharmacological and antimicrobial studies on different tea-tree oils (*Melaleuca alternifolia, Leptospermum scoparium* or Manuka and *Kunzea ericoides* or Kanuka), originating in Australia and New Zealand. Phytother. Res..

[8] Dellacassa E., Lorenzo D., Mondello L., Cotroneo A.A.  (1997). Uruguayan essential oils. Part VII. Composition of leaf oil of *Eugenia uruguayensis* Camb. Var uruguayensis (Myrtaceae). J. Essent. Oil Res..

[9] Bello A., Rodriguez M.L., Cariñeiras N., Urquiola A., Rosado A., Pino J.A. (1995). Major components of the leaf oil of *Eugenia banderesis* Urb. J. Essent. Oil Res..

[10] Martins R.C.C., Alegrio L.V., Castro R.N., Godoy R.L.O. (1999). Constituents of the essential oil of *Eugenia nítida* Camb. (Myrtaceae). J. Essent. Oil Res..

[11] Lima N.R., Cerqueira S.H.F., Favero O.A., Romoff P., Lago J.H.G. (2008). Composition and chemical variation of the essential oil from levaes of *Eugenia brasiliensis* Lam. and *Eugenia* sp. J. Essent. Oil Res..

[12] Weyerstahl P., Marschall-Weyerstahl H., Christiansen C., Oguntimein B.O., Adeoye A.O. (1988). Volatile constituents of *Eugenia uniflora* leaf oil. Planta Med..

[13] Chang R., de Morais S.A.L., Napolitano D.R., Duarte K.C., Guzman V.B., do Nascimento E.A. (2011). A new approach for quantifying furanodiene and curzerene. A case study on the essential oils of *Eugenia uniflora* L., Myrtaceae (Pitangueira) leaves. Rev. Bras. Farmacogn..

[14] Gallucci S., Neto A.P., Porto C., Barbizan D., Costa I., Marques K., Benevides P., Figueiredo R.  (2010). Essential oil of *Eugenia uniflora* L.: An industrial perfumery approach. J. Essent. Oil Res..

[15] Lorenzi H., Matos F.J.A. (2002). Plantas Medicinais no Brasil: Nativas e Exóticas Cultivadas.

[16] Auricchio M.T., Bacchi E.M. (2003). Folhas de *Eugenia uniflora* L. (pitanga): Propriedades farmacobotânicas, químicas e farmacológicas. Rev. Inst. Adolfo Lutz.

[17] Apel M.A., Sobral M., Zuanazzi J.A., Henriques A.T. (2006). Essential oil composition of four *Plinia* species (Myrtaceae). Flavour Fragrance J..

[18] Duarte A.R., Santos S.C., Seraphin J.C., Ferri P.H. (2010). Environmental influence on phenols and essential oils of *Myrciaria cauliflora* leaves. J. Braz. Chem. Soc..

[19] Bragança L.A.R. (1996). Plantas Medicinais Antidiabéticas. Uma Abordagem Multidisciplinar.

[20] Souza-Moreira T.M., Moreira R.R.D., Sacramento L.V.S., Pietro R.C.L.R.  (2010). Histochemical, phytochemical and biological screening of *Plinia cauliflora* (Mart.) Kausel (Myrtaceae) leaves. Rev. Bras. Farmacogn.

[21] Sartorelli P., Marquioreto A.D., Lima M.E.L., Amaral-Baroli A., Moreno P.R.H. (2007). Chemical composition and antimicrobial activity of the essential oils from two species of *Eucalyptus*. Phytother. Res..

[22] Lago J.H.G., Carvalho L.A.C., da Silva F.S., Toyama D.D., Favero O.A., Romoff P. (2010). Chemical composition and anti-inflammatory evaluation of essential oils from leaves and stem barks from *Drimys brasiliensis* Miers (Winteraceae). J. Braz. Chem. Soc..

[23] Urbiego G., Taher H.A., Talenti E.C. (1987). Chemical composition of essential oil of *Eugenia uniflora*. An. Asoc. Quim. Argent..

[24] Maia J.G.S., Andrade M.H.L., da Silva M.H.L., Zoghbi M.G.B. (1999). A new chemotype of *Eugenia uniflora* L. from North Brazil. J. Essent. Oil Res..

[25] Ogunwande I.A., Olawore N.O., Ekundayo O., Walker T.M., Schmidt J.M., Setzer W.N. (2005). Studies on the essential oils composition, antibacterial and cytotoxicity of *Eugenia uniflora* L. Int. J. Aromather..

[26] Melo R.M., Corrêa F.S.V., Amorim A.C.L., Miranda A.L.P., Rezende C.M.  (2007). Identification of impact aroma compounds in *Eugenia uniflora* L. (Brazilian pitangueira) leaf essential oil. J. Braz. Chem. Soc..

[27] El-Shabrawy A.O. (1995). Essential oil composition and tannin contents of the leaves of *Eugenia uniflora* L. grown in Egypt. Bull. Fac. Pharm. Cairo Univ..

[28] Morais S.M., Craveiro A.A., Machado M.I.L., Alencar J.W., Matos J.A. (1996). Volatiles constituents of *Eugenia uniflora* leaf oil from Northeastern Brazil. J. Essent. Oil Res..

[29] Costa D.P., Santos S.C., Seraphin J.C., Ferri P.H. (2009). Seasonal variability of essential oils of *Eugenia uniflora* leaves. J. Braz. Chem. Soc..

[30] Espinel-Ingroff A. (2003). *In vitro* antifungal activities of anidulafungin and micafungin, licensed agents and the investigational triazole posaconazole as determined by NCCLS methods for 12,052 fungal isolates: Review of the literature. Rev. Iberoam. Micol..

[31] Marino I.R., Panarello G., Singh N. (2002). Efficacy of Aspergillus galactomannan-directed preemptive therapy for the prevention of invasive aspergillosis in organ transplant recipients. Transpl. Infect. Dis..

[32] Chorianopoulos N., Kalpoutzakis E., Aligiannis N., Mitaku S., Nychas G.J., Haroutounian S.A. (2004). Essential oils of *Satureja*, *Origanum*, and *Thymus* species: Chemical composition and antibacterial activities against foodborne pathogens. J. Agric. Food Chem..

[33] Gutierrez J., Rodriguez G., Barry-Ryan C., Bourke P. (2008). Efficacy of plant essential oils against foodborne pathogens and spoilage bacteria associated with ready-to-eat vegetables: Antimicrobial and sensory screening. J. Food Prot..

[34] Tiwari B.K., Valdramidis V.P., O’Donnell C.P., Muthukumarappan K., Bourke P., Cullen P.J. (2009). Application of natural antimicrobials for food preservation. J. Agric. Food Chem..

[35] Sokmen A., Vardar-Unlu G., Polissiou M., Daferera D., Sokmen M., Donmez E.  (2003). Antimicrobial activity of essential oil and methanol extracts of *Achillea sintenisii* Hub. Mor. (Asteraceae). Phytother. Res..

[36] Blanco J.L., Garcia M.E. (2008). Immune response to fungal infections. Vet. Immunol. Immunopathol..

[37] Nielsen K., Heitman J. (2007). Sex and virulence of human pathogenic fungi. Adv. Genet..

[38] Tamae C., Liu A., Kim K., Sitz D., Hong J., Becket E., Bui A., Solaimani P., Tran K.P., Yang H., Miller J.H. (2008). Determination of antibiotic hypersensitivity among 4,000 single-gene-knockout mutants of *Escherichia coli*. J. Bacteriol..

[39] Adams R.P. (1995). Identification of Essential Components by Gas Chromatography/Mass Spectroscopy.

